# Reading, writing, and repair: the role of ubiquitin and the ubiquitin-like proteins in DNA damage signaling and repair

**DOI:** 10.3389/fgene.2013.00045

**Published:** 2013-04-01

**Authors:** Jordan B. Pinder, Kathleen M. Attwood, Graham Dellaire

**Affiliations:** ^1^Department of Pathology, Dalhousie UniversityHalifax, NS, Canada; ^2^Department of Biochemistry and Molecular Biology, Dalhousie UniversityHalifax, NS, Canada

**Keywords:** ubiquitin, SUMO, DNA repair, E3 ligase, RNF8, MDC1, H2AX

## Abstract

Genomic instability is both a hallmark of cancer and a major contributing factor to tumor development. Central to the maintenance of genome stability is the repair of DNA damage, and the most toxic form of DNA damage is the DNA double-strand break. As a consequence the eukaryotic cell harbors an impressive array of protein machinery to detect and repair DNA breaks through the initiation of a multi-branched, highly coordinated signaling cascade. This signaling cascade, known as the DNA damage response (DDR), functions to integrate DNA repair with a host of cellular processes including cell cycle checkpoint activation, transcriptional regulation, and programmed cell death. In eukaryotes, DNA is packaged in chromatin, which provides a mechanism to regulate DNA transactions including DNA repair through an equally impressive array of post-translational modifications to proteins within chromatin, and the DDR machinery itself. Histones, as the major protein component of chromatin, are subject to a host of post-translational modifications including phosphorylation, methylation, and acetylation. More recently, modification of both the histones and DDR machinery by ubiquitin and other ubiquitin-like proteins, such as the small ubiquitin-like modifiers, has been shown to play a central role in coordinating the DDR. In this review, we explore how ubiquitination and sumoylation contribute to the “writing” of key post-translational modifications within chromatin that are in turn “read” by the DDR machinery and chromatin-remodeling factors, which act together to facilitate the efficient detection and repair of DNA damage.

## INTRODUCTION

Genomic stability is continuously being threatened by insults arising from both endogenous (metabolic) and exogenous (environmental) sources (Panier and Durocher, 2009; van Attikum and Gasser, 2009). The result can be a variety of DNA lesions including damaged or modified bases, intra-strand cross-links, as well as single- and double-strand DNA breaks (Ciccia and Elledge, 2010). DNA double-strand breaks (DSBs) represent one of the most cytotoxic DNA lesions (Wyman and Kanaar, 2006). DSBs can be produced during normal cellular metabolism and DNA replication, as well as exogenously through exposure to ionizing radiation (IR) or chemical mutagens (Ciccia and Elledge, 2010). DNA DSBs are repaired either by non-homologous end-joining (NHEJ), which occurs at any time in the cell cycle, or by homologous recombination (HR), which occurs predominately in S and G2 phase, peaking in mid-S phase (Karanam et al., 2012). If not properly repaired, DNA DSBs can lead to a spectrum of mutations that can trigger cell death if normal checkpoint function is intact, or induce cellular transformation by activating oncogenes or disrupting tumor suppressor function (Wyman and Kanaar, 2006).

**FIGURE 1:**
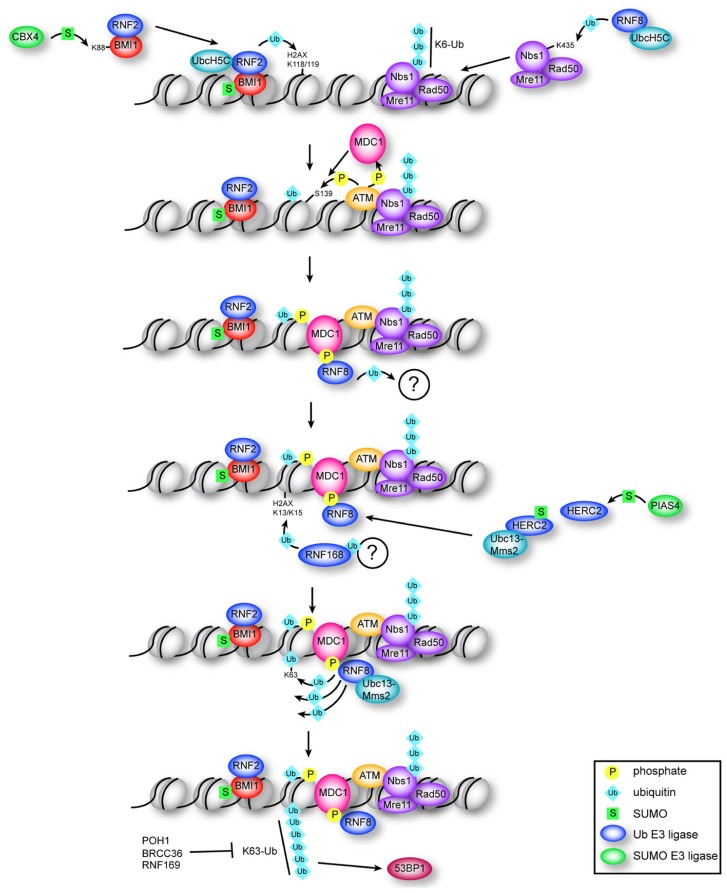
**Multiple roles for ubiquitin and SUMO in the early DDR**. Constitutive ubiquitination of NBS1 by RNF8–UbcH5c is required for localization of the MRN complex to DNA breaks. BMI1–RNF2 is targeted to DSBs by damage-induced CBX4-mediated sumoylation of BMI1. RNF2–BMI1–UbcH5c monoubiquitinates H2AX on K118/K119, which is required for recruitment of ATM and efficient production of γ-H2AX. MDC1 binds γ-H2AX and is phosphorylated by ATM, which recruits RNF8. RNF8 ligase activity is required (through an unknown mechanism) to recruit RNF168, which ubiquitinates H2AX at K13/K15. RNF8 catalyzes K63 chains on K13/K15-ubiquitinated H2AX through association with Ubc13–Mms2, which depends on interaction of RNF8 with sumoylated HERC2. Formation of K63 chains promotes recruitment of 53BP1 through an unknown mechanism, and is antagonized by RNF169 and by the DUBs POH1 and BRCC36.

As a consequence, to maintain genomic stability a multi-branched, highly coordinated signaling cascade is initiated following the induction of even a single DNA DSB (Huang et al., 1996). This signaling cascade, termed the DNA damage response (DDR) integrates several cellular responses including DNA repair, cell cycle checkpoint activation, transcriptional regulation, or apoptosis if damage proves too severe (Bao, 2011). One of the hallmarks of the cellular response to DNA DSBs is the focal accumulation of many of the DDR proteins at the break site (van Attikum and Gasser, 2009). This assembly of repair factors on DNA DSBs occurs in a highly regulated manner according to a strict hierarchy and is reliant on the phosphorylation of the key histone variant H2AX (termed γ-H2AX; **Figure 1**; Rogakou et al., 1998; Paull et al., 2000). Following DNA DSB induction, H2AX is rapidly phosphorylated by a set of phosphoinositide-3-kinase-related kinases: ATM (ataxia telangiectasia mutated), ATR (ATM- and RAD3-related), and DNA-PK (DNA-dependent protein kinase; Ward and Chen, 2001; Stiff et al., 2004) and is crucial for rapid amplification of the DNA damage signal. MDC1 (mediator of DNA damage checkpoint 1), a key mediator of the DDR, binds directly to γ-H2AX and recruits the MRE11/RAD50/NBS1 (MRN) complex to break sites (Lukas et al., 2004; Stucki et al., 2005). The MRN complex in turn can further stimulate ATM activity leading to rapid spreading of γ-H2AX around the DNA break, and therefore the amplification of the DDR signal (Uziel et al., 2003; Lee and Paull, 2005). In addition, γ-H2AX is crucial for the effective recruitment and retention of many DNA repair enzymes at DNA DSBs, including 53BP1, BRCA1, and RAD51 (Paull et al., 2000; Nakamura et al., 2010) as well as chromatin-remodeling complexes such as SWR1 and INO80 (Downs et al., 2004; Morrison et al., 2004; van Attikum et al., 2004), resulting in the accumulation of a high concentration of repair factors in the vicinity of a break. The recruitment of these factors to the site of DNA DSBs is complicated by the fact that the physiological substrate upon which repair must occur is not naked DNA, but rather DNA complexed with histone proteins in the form of chromatin. Furthermore, the compaction of eukaryotic chromatin is variable, with DNA being packaged as either euchromatin or heterochromatin. Euchromatin represents loosely packed, transcriptionally active gene-rich regions, while heterochromatin is generally characterized by highly repetitive regions that are tightly compacted and are transcriptionally silent (Gelato and Fischle, 2008). The differential compaction of DNA into either euchromatin or heterochromatin thus serves to control access of various proteins to the underlying DNA, regulating key cellular processes such as transcription, DNA replication, and repair (Groth et al., 2007; Li et al., 2007). Accordingly, the interplay between chromatin and DNA repair factors plays a central role in the cellular response to DSBs, and modulation of chromatin structure is critical for mediating access of repair proteins to underlying DNA lesions (Costelloe et al., 2006). To overcome the physical barrier posed by chromatin structure, a variety of histone modifying enzymes and chromatin-remodeling complexes are recruited to break sites following DNA damage to facilitate binding of DNA repair proteins (Dinant et al., 2008). Histones are also subject to a vast array of post-translational modifications including phosphorylation, methylation, acetylation, ubiquitination, and sumoylation. Together, these modifications can influence the structure of chromatin directly, for example by impacting the stability of individual nucleosomes, or indirectly by creating or eliminating binding sites for non-histone proteins, such as ATP-dependent chromatin remodelers that can in turn facilitate changes in chromatin organization (Saha et al., 2006).

Whereas the influence of acetylation, methylation, and phosphorylation on chromatin structure and the impact of these modifications on DNA repair has been extensively investigated, we are only now beginning to appreciate that a much larger spectrum of protein modifications is at play during the DDR. In particular, the modification of both chromatin and DNA repair factors by ubiquitin and the small ubiquitin-like modifiers (SUMOs) has recently been shown to play a central role in the detection and repair of DNA DSBs. Here we will explore how ubiquitination and sumoylation control key post-translational modifications within chromatin that are recognized by DNA repair and chromatin-remodeling factors, which act together to facilitate the efficient detection and repair of DNA damage (summarized in **Figure 1**).

## THE UBIQUITIN-LIKE FAMILY OF PROTEINS: MODULATING ASSEMBLY OF PROTEIN COMPLEXES THROUGH COVALENT AND NON-COVALENT INTERACTIONS

Ubiquitin and SUMO are two members of a family of ubiquitin-like proteins (UBLs) that are conjugated to target proteins post-translationally (Hochstrasser, 2009). Ubiquitin and SUMO can be attached to lysine residues in target proteins through an isopeptide bond, and also bind non-covalently to interacting partners at specific domains called ubiquitin-binding domains (UBDs) and SUMO-interaction motifs (SIMs), respectively.

The conjugation systems for ubiquitin and SUMO are mediated by a set of enzymes specific for each UBL (Johnson and Blobel, 1997). The mechanism of ubiquitin conjugation is summarized below. Carboxy-terminal residues in the primary translation product of ubiquitin are removed by specific proteases to expose a diglycine motif that is ultimately linked to a nucleophilic side chain (usually lysine) in the target protein. Catalysis occurs in a sequential manner by three distinct classes of enzymes: an activating enzyme (E1), conjugating enzyme (E2), and ligase (E3). Ubiquitin is first activated in an ATP-driven reaction that forms a high-energy thioester bond between its terminal carboxylate and a cysteine residue in the E1. Ubiquitin is transferred via transthioesterification to the active site cysteine residue of the E2, and then is generally conjugated to a lysine residue in the target protein with the assistance of an E3 ligase. Ubiquitin E3 ligases are divided into two families, the largest of which is the really interesting new gene (RING) E3 family, for which there are more than 600 potential members in mammals (Li et al., 2008). RING domain ligases bridge the interaction between E2-ubiquitin conjugates and the target protein, providing an orientation favorable to catalysis. The smaller family of ubiquitin E3s (~30 in mammals; Metzger et al., 2012) are the HECT (homologous to the E6AP carboxyl terminus) ligases, through which an additional thioester intermediate is formed during transfer of ubiquitin to the substrate (Rotin and Kumar, 2009).

Sumoylation occurs by a similar mechanism as ubiquitination, with some notable distinctions. Mammals encode ~40 ubiquitin E2s, but only one SUMO E2, Ubc9 (Kerscher et al., 2006; Gareau and Lima, 2010). Several types of SUMO E3s have been characterized to date, one family containing an SP-RING domain that is analogous to the RING domain of ubiquitin E3s. Covalent attachment of ubiquitin and SUMO to target proteins is reversible, and removal is catalyzed by de-ubiquitinating enzymes (DUBs) and SUMO-specific proteases (sentrin-specific proteases, SENPs), respectively. Although vertebrates encode just a single ubiquitin protein, there are at least three major isoforms of SUMO that are relevant for DNA repair in mammals, encoded by separate genes, SUMO-1, SUMO-2, and SUMO-3 (Citro and Chiocca, 2013). There is also evidence of a fourth SUMO paralog in humans called SUMO-4; however, it appears to function in the cytoplasm and its expression is limited to kidney, spleen, and lymph tissue (Bohren et al., 2004; Guo et al., 2004). Due to the nearly indistinguishable function and close similarities in sequence between SUMO-2 and -3 (~97% identical), they are commonly referred to as SUMO-2/3 in the literature (Bayer et al., 1998). Ubiquitin is a target of itself, and can form branched chains at any of its seven lysine residues (K6, K11, K27, K29, K33, K48, K63) and linear chains through its amino-terminal methionine amino group (Husnjak and Dikic, 2012; Walczak et al., 2012). The most well-known function of ubiquitin is to target proteins for proteasomal degradation, which is signaled by K48-linked chains. SUMO-1 is mostly associated with mono-sumoylation whereas SUMO-2 and -3, like ubiquitin, can form poly-SUMO chains via K11, with SUMO-2 forming chains more readily than SUMO-3, and SUMO-1 potentially acting as a SUMO chain terminator (Tatham et al., 2001).

Although ubiquitination is typically associated with proteasomal degradation, both ubiquitin and SUMO conjugation can serve to modulate the interacting partners of the modified protein, in many cases by enabling recognition by proteins containing UBDs and SIMs, respectively. UBDs have many different subtypes, with those relevant in DDR-pathway proteins including MIU (motif interacting with ubiquitin), UIM (ubiquitin interacting motif), and UMI (UIM and MIU-related UBD; Hicke et al., 2005; Dikic et al., 2009; Pinato et al., 2011; Husnjak and Dikic, 2012). Most SIMs are characterized by a hydrophobic core often flanked by acidic residues (Song et al., 2004; Hannich et al., 2005; Hecker et al., 2006). Specificity of UBD-containing proteins can be conferred by tandem UBDs that recognize a specific ubiquitin chain topology, and also by additional peptide motifs to which the UBDs are juxtaposed (Husnjak and Dikic, 2012; Panier et al., 2012).

Ubiquitin and SUMO have critical functions in DNA repair, and protein conjugates of ubiquitin and SUMO are observed at sites of DNA DSBs (Galanty et al., 2009; Morris et al., 2009; Stewart et al., 2009). Both K48- and K63-linked ubiquitin chains are detected at DSBs immediately after damage, although K63-linked chains persist for a much longer time (Feng and Chen, 2012). Ubiquitin conjugates are observed as soon as 15 s following DNA damage. This initial wave is mediated by the ubiquitin E3 ligase cased hole formation resistivity (CHFR), that binds to poly(ADP-ribosyl)ated proteins, which rapidly accumulate at DNA breaks (Liu et al., 2012). A second wave of ubiquitination occurs about one minute after damage, and is mediated by the E3 ring finger protein 8 (RNF8; discussed below; Liu et al., 2012). SUMO-1 and SUMO-2/3 are also observed at breaks immediately after damage, though SUMO-1 accrual may lag slightly behind SUMO-2/3 (Galanty et al., 2009; Hu et al., 2012). SUMO persists at breaks for several hours after damage (Galanty et al., 2009). Ubiquitin and SUMO serve to recruit and assemble repair factors at sites of DNA damage through interaction with UBDs and SIMs, respectively. Recent advances in the function of ubiquitin and SUMO during the repair of DNA DSBs will be discussed in more detail below, and key substrates of sumoylation and ubiquitination involved in DNA DSB repair are summarized in **Tables 1** and **2**, respectively.

**Table 1:** Sumoylation targets in the early DDR.

SUMO target	Isoform	Site(s)	E3(s)	Proposed function	Reference
BMI1	SUMO-1	K88	CBX4	Accumulation of BMI1 at DSBs	Ismail et al. (2012)
HERC2	SUMO-1	nd	PIAS4	Promotes binding to RNF8	Danielsen et al. (2012)
RNF168	SUMO-1	nd	PIAS4	Maintain RNF168 levels	Danielsen et al. (2012)
53BP1	SUMO-1	nd	PIAS4	Unknown	Galanty et al. (2009)
BRCA1	SUMO-1, SUMO-2/3	nd	PIAS1, PIAS4	Stimulates ligase activity	Galanty et al. (2009); Morris et al. (2009)
MDC1	SUMO-1, SUMO-2/3	K1840	PIAS4	Signal for RNF4-mediated ubiquitination	Luo et al. (2012)
RAP80	SUMO-1, SUMO-3	nd	nd	unknown	Yan et al. (2007)
RPA70	SUMO-2/3	K449, K577	nd	Facilitates RAD51 recruitment	Dou et al. (2010)

*nd, not determined.*

**Table 2:** Ubiquitination targets in the early DDR.

Ubiquitin target	Type of linkage	Site(s)	E2	E3	Proposed function	Reference
PARP1	K48-Ub chains, K63-Ub chains	K88	UbcH5C Ubc13	CHFR	Displacement of PARP1 from DSB sites	Liu et al. (2012)
H2AX	Mono-Ub	K119, K120	UbcH5C	RNF2–BMI1	Required for recruitment of ATM	Facchino et al. (2010); Ismail et al. (2010), Bentley et al. (2011); Ginjala et al. (2011), Wu et al. (2011)
H2AX	Mono-Ub, some K63-Ub chains	K13, K15	UbcH5C	RNF168	Priming for RNF8-mediated ubiquitination	Gatti et al. (2012); Mattiroli et al. (2012)
Ub-H2AX (K13/15)	K63-Ub chains	K13, K15	Ubc13	RNF8	Important for 53BP1 recruitment	Mattiroli et al. (2012)
MDC1	K63-Ub chains	K1977	Ubc13	nd	Recruits RAP80	Strauss et al. (2011)
SUMO-MDC1 (K1840)	K48-Ub chains	nd	nd	RNF4	Degradation of MDC1	Luo et al. (2012)
NBS1	K6-Ub chains	K435	UbcH5C	RNF8	Recruits NBS1 to DSBs	Lu et al. (2012)
JMJD2A	K48-Ub chains	nd	UbcH5C	RNF8/RNF168	Proteasomal degradation, to expose H4K20me2	Mallette et al. (2012)

*nd, not determined.*

## MONOUBIQUITINATION OF H2AX BY RNF2: AN EARLY STEP IN DNA REPAIR

One of the earliest events in DSB repair is the recruitment of ATM kinase to the site of the break, where it phosphorylates numerous targets, in particular histone H2AX at S139 to form γ-H2AX. Recent reports illustrate a role for the E3 ubiquitin ligase RNF2 (RING1b/RING2) in ATM recruitment. RNF2 and its adaptor protein BMI1 (B lymphoma Mo-MLV insertion region 1 homolog) are RING domain-containing proteins of the Polycomb repressive complex 1 (PRC1) (Sparmann and van Lohuizen, 2006) and catalyze monoubiquitination of histone H2A (Wang et al., 2004; Cao et al., 2005; Buchwald et al., 2006). Approximately 5–15% of H2A is constitutively monoubiquitinated (Levinger and Varshavsky, 1980; West and Bonner, 1980) and serves to repress transcription through inhibition of RNA polymerase II transcription elongation (Zhou et al., 2008). RNF2–BMI1 was also shown to play a role in DNA repair, based on observations that depletion of either RNF2 or BMI1 causes increased sensitivity to IR, and a delayed DDR (Facchino et al., 2010; Ismail et al., 2010; Ginjala et al., 2011; Pan et al., 2011; Wu et al., 2011). Following DNA damage, RNF2–BMI1 catalyzes monoubiquitination of H2AX at K119 and K120 (K118 and K119 in H2A; Bergink et al., 2006; Ginjala et al., 2011; Pan et al., 2011; Wu et al., 2011). This modification is required for recruitment of ATM to sites of damage, and consequently, is necessary for efficient formation of γ-H2AX (Pan et al., 2011; Wu et al., 2011). Since the kinase DNA-PK is functionally redundant to ATM in phosphorylation of H2AX (Stiff et al., 2004), knock-down of RNF2 in the presence of a DNA-PK inhibitor is required to completely ablate formation of γ-H2AX (Pan et al., 2011).

BMI1 tethers RNF2 to DNA, and associates more stably with damaged compared to undamaged chromatin (Ismail et al., 2010). Computational models based on a recently derived crystal structure of BMI1–RNF2–ubiquitin-conjugating enzyme H5c (UbcH5c) suggest that the complex binds to both nucleosomal DNA and histone H4 (Bentley et al., 2011), while initial recruitment of RNF2–BMI1 to DSBs is dependent on sumoylation of BMI1 (Ismail et al., 2012). The PRC1 complex member CBX4 (chromobox homolog 4) promotes sumoylation (SUMO-1) of BMI1 at K88, with the BMI1 K88R mutant failing to be recruited to repair foci (Ismail et al., 2012). Although BMI1 is required for initial recruitment of ATM, ATM is required for sustained localization of BMI1 at breaks, which is important for efficient HR (Ginjala et al., 2011). Further experimentation will be required to elucidate the mechanism by which sumoylation mediates RNF2–BMI1 assembly at DSBs, and how H2AX ubiquitination enables recruitment of ATM. Initial studies indicate that ubiquitination of H2A may weaken interaction with DNA, destabilizing the nucleosome (Li et al., 1993). Consistent with this hypothesis, K118 and K119 in H2A form hydrogen bonds with DNA that would be disrupted by conjugation to ubiquitin (Biswas et al., 2011). However, nucleosome stability has yet to be directly implicated in recruitment of ATM.

## MULTIPLE CATALYTIC ROLES FOR THE UBIQUITIN E3 LIGASE RNF8 IN DNA REPAIR

While H2A monoubiquitination is an important early step in the DDR, extensive ubiquitin chains linked at K48 and K63 are also observed in the vicinity of DNA breaks (Meerang et al., 2011). K63 chains are particularly important in recruitment of downstream DDR repair proteins, such as RAP80 and 53BP1 (Ciccia and Elledge, 2010; Polo and Jackson, 2011; Hu et al., 2012). The major E3 ligase responsible for catalyzing formation of these chains is RNF8. Following formation of γ-H2AX, ATM phosphorylates MDC1, creating a binding site for the forkhead-associated (FHA) domain of RNF8 (Huen et al., 2007; Mailand et al., 2007; Marteijn et al., 2009). RNF8 is required for recruitment of another E3 ubiquitin ligase, RNF168, to repair foci (Doil et al., 2009; Stewart et al., 2009). RNF8 and RNF168 act in concert to catalyze non-proteolytic K63-linked ubiquitin chains conjugated to H2AX on residues K13 and K15 (Gatti et al., 2012; Mattiroli et al., 2012). These residues are located on the opposite side of the nucleosome as the sites targeted for monoubiquitination by RNF2. Polyubiquitination of H2AX is required for proper DNA DSB signaling, as expression of ligase-dead RNF168 affects recruitment of downstream DDR repair factors, including RAP80, BRCA1, and 53BP1 (Mattiroli et al., 2012).

**FIGURE 2:**
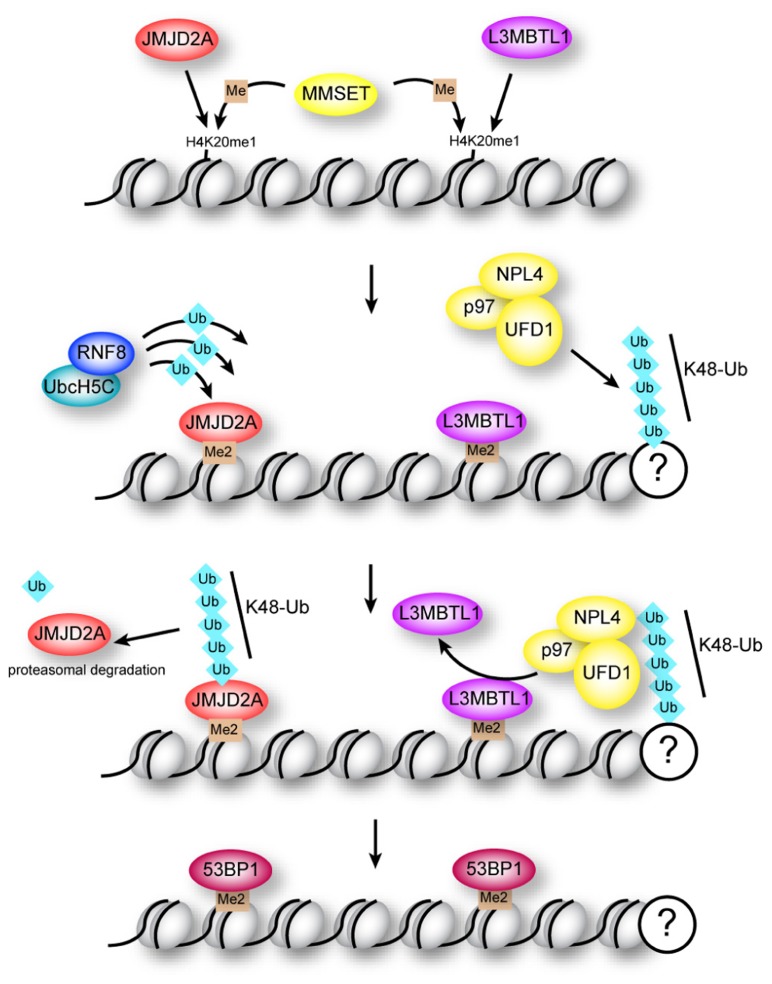
**Regulation of 53BP1 association with H4K20me2 at sites of DNA damage**. The methyltransferase MMSET promotes di-methylation of mono-methylated lysine 20 in histone H4 (H4K20me1) at DNA breaks. Binding of 53BP1 to H4K20me2 is opposed by methyllysine-binding proteins JMJD2A and L3MBTL1 in two potentially overlapping pathways. Proteasomal degradation of JMJD2A is triggered by RNF8-dependent ubiquitination, while ejection of L3MBTL1 is mediated by the ubiquitin-dependent segregase p97.

Despite the preliminary *in vitro* biochemical evidence pointing to RNF8 mediating the initial “priming” ubiquitination of H2AX followed by RNF168 during the DDR, new evidence has come to light that challenges this hierarchy in the establishment of the K63 ubiquitin chains on H2AX. Although RNF8 can ubiquitinate free H2A *in vitro*, and despite the fact that RNF168 recruitment to DNA breaks requires both the catalytic activity of RNF8, as well as the MIU domains of RNF168 (Doil et al., 2009; Stewart et al., 2009), nucleosomal H2A is a substrate of RNF168 and cannot be modified by RNF8 (Gatti et al., 2012; Mattiroli et al., 2012). Thus, RNF8 efficiently adds K63-linked ubiquitin chains to H2A following initial ubiquitination by RNF168 (Mattiroli et al., 2012). Therefore, the requirement of RNF8 catalytic activity for RNF168 recruitment may reflect the contribution of the RNF8-mediated ubiquitination of a non-nucleosomal protein (Mattiroli et al., 2012). While this protein has not yet been identified, RNF8 has been shown to target other DDR-pathway proteins for ubiquitination, including the MRN component NBS1 (Lu et al., 2012), and JMJD2A, which obstructs binding of 53BP1 to dimethylated K20 in histone H4 (H4K20me2; Mallette et al., 2012; **Figure 2**, and discussed below). Ubiquitination of NBS1 is required for recruitment of both NBS1 and MRE11 to DNA DSBs, and deficient NBS1 ubiquitination impairs the HR repair pathway (Lu et al., 2012).

Supporting the hypothesis that RNF8 is responsible for H2AX polyubiquitination, RNF8 interacts with the E2 Ubc13–Mms2, the only E2 capable of forming K63-linked ubiquitin chains (Hofmann and Pickart, 1999; VanDemark et al., 2001; Eddins et al., 2006; Plans et al., 2006). Like many ubiquitin E3s, RNF8 can interact with multiple E2s, and preferential assembly of RNF8 with Ubc13–Mms2 at DNA repair centers is mediated by the HECT E3 ligase HERC2 (Bekker-Jensen and Mailand, 2010). Association of RNF8 with Ubc13–Mms2 does not appear to strictly require the catalytic activity of HERC2, but rather is promoted through interaction of HERC2 with RNF8, an interaction in turn regulated by phosphorylation and sumoylation of HERC2 (Bekker-Jensen and Mailand, 2010; Danielsen et al., 2012). HERC2 is a target of the SUMO E3 ligase PIAS4 (protein inhibitor of activated STAT protein 4), and also contains a novel ZZ zinc finger SUMO-binding domain (Danielsen et al., 2012). HERC2 is dependent on both sumoylation and its SUMO-binding domain for interaction with RNF8, suggesting that an intramolecular SUMO–SIM interaction may induce a conformational change in HERC2 to enable binding to RNF8, stabilizing RNF8–Ubc13 association (Danielsen et al., 2012).

PIAS4 also mediates sumoylation of RNF168, which may be important for maintaining sufficient RNF168 protein levels, since depletion of PIAS4 leads to decreased RNF168 half-life, and decreased transcript levels (Danielsen et al., 2012).

## NEGATIVE REGULATION OF UBIQUITIN SIGNALING AT DNA BREAKS: SHIFTING THE BALANCE OF DNA DSB REPAIR PATHWAYS

Antagonizing the recruitment of UBD-containing repair proteins to sites of DNA damage is also an important regulatory mechanism in repair of DNA DSBs, and may function in shifting the balance between DNA DSB repair by NHEJ versus HR. Recent studies uncovered three distinct mechanisms for antagonizing ubiquitin-dependent protein recruitment to DSBs: (1) turnover of ubiquitin chains by DUBs, (2) ejection of ubiquitinated proteins by an ubiquitin-directed segregase, and (3) competition by an RNF168 paralog.

The NHEJ pathway is promoted by assembly of 53BP1 at DNA repair centers (Escribano-Diaz et al., 2013; Zimmermann et al., 2013). Formation of K63 ubiquitin chains on H2AX promotes recruitment of 53BP1 to repair foci through a currently unknown mechanism (Hartlerode et al., 2012). Efficient recruitment of 53BP1 to breaks is also dependent on interaction of the Tudor domains in 53BP1 with H4K20me2 (**Figure 2**). This constitutive H4 modification is enriched at breaks due to the H4 methyltransferase MMSET, which localizes to breaks following damage (Pei et al., 2011). However, two proteins, JMJD2A and L3MBTL1, appear to have a common function in obstructing access of 53BP1 to histone H4 (Acs et al., 2011; Meerang et al., 2011; Mallette et al., 2012). Importantly, association of these proteins with chromatin is regulated by ubiquitination through two distinct pathways (**Figure 2**; Acs et al., 2011; Meerang et al., 2011; Mallette et al., 2012).

JMJD2A is a Tudor domain-containing protein that binds H4K20me2 with higher affinity than 53BP1 (Mallette et al., 2012). Accessibility of 53BP1 to H4K20me2 is enabled through proteasomal degradation of JMJD2A triggered by RNF8/RNF168-mediated K48-linked ubiquitin chains (Mallette et al., 2012). This study demonstrated that assembly of RNF8 with UbcH5c enables it to catalyze K48-linked chains, highlighting the importance of RNF8 in catalyzing both K48- and K63-linked ubiquitin at DNA repair centers.

L3MBTL1 is a Polycomb protein that binds H4K20me2 through multiple MBT domains, and is ejected from these sites by the “molecular corkscrew” activity (Ramadan, 2012) of the AAA-ATPase p97/VCP (valosin-containing protein; Acs et al., 2011; Meerang et al., 2011). The p97–UFD1–NLP4 complex has ubiquitin-dependent segregase activity, and requires RNF8 for turnover of K48-linked ubiquitin chains at DNA breaks (Acs et al., 2011; Meerang et al., 2011). One of the functions of this segregase activity is to displace L3MBTL1 from chromatin at DNA breaks, unmasking the binding site for 53BP1 (Acs et al., 2011; Meerang et al., 2011).

Attenuation of ubiquitin signaling at DNA breaks is also regulated by two members of the JAMM/MPN+ family of DUBs that specifically hydrolyze K63-linked ubiquitin chains: the BRCA1-A complex member BRCC36, and the 19S proteasomal lid subunit POH1 (Cooper et al., 2009; Shao et al., 2009; Butler et al., 2012). Cells deficient in BRCC36 or POH1 are sensitized to IR, implicating a role for proteolysis of K63 chains in the DDR (Shao et al., 2009; Butler et al., 2012). BRCC36 and POH1 antagonize the actions of RNF8/RNF168, hydrolyzing the K63 linkages that promote 53BP1 recruitment. POH1 promotes association of JMJD2A with chromatin, and therefore suppresses 53BP1 recruitment and the NHEJ pathway (Butler et al., 2012). POH1 also appears to promote HR by a mechanism independent of 53BP1 (Butler et al., 2012). In cells with deficient RNF8/RNF168 activity, formation of 53BP1 foci and NHEJ pathway utilization can be restored by co-depletion of POH1 (Butler et al., 2012).

Accumulation of K63 ubiquitin chains at DNA repair centers is also antagonized through RNF169-mediated competition with UBD-containing proteins for binding sites at DNA DSBs. Through bioinformatics analyses, three groups independently identified RNF169 as a paralog of RNF168, suggesting potential involvement of RNF169 in the DDR signaling cascade (Chen et al., 2012; Panier et al., 2012; Poulsen et al., 2012). Following DNA damage, RNF169 is targeted to repair foci through one of its two UBDs, MIU2 (Chen et al., 2012; Panier et al., 2012; Poulsen et al., 2012). RNF168 is also required for accumulation of RNF169 at repair foci (Chen et al., 2012; Panier et al., 2012; Poulsen et al., 2012). Although purified RNF169 displays E3 ligase activity, unlike RNF168 it is inactive toward H2A (Poulsen et al., 2012). Instead, RNF169 inhibits recruitment of proteins that depend on RNF8/RNF168 activity for recruitment to repair foci. Over-expression of RNF169 out-competes RNF168 for association with chromatin, leading to a reduction in ubiquitinated proteins at breaks, and impairing 53BP1 accrual at DNA repair foci, causing a delayed DDR (Chen et al., 2012; Panier et al., 2012; Poulsen et al., 2012). Consistently, depletion of RNF169 leads to prolonged DDR signaling and a sustained G_2_/M checkpoint after damage (Chen et al., 2012).

What is the functional significance of opposing RNF8/RNF168-dependent K63 ubiquitination? One emerging hypothesis is that K63 signaling mediates 53BP1 assembly at DNA breaks, promoting the NHEJ pathway (**Figure 1**), since knock-down of RNF168 selectively affects NHEJ (Meerang et al., 2011; Poulsen et al., 2012). 53BP1 and RAP80 seem to suppress HR-mediated repair (Bothmer et al., 2010; Coleman and Greenberg, 2011; Hu et al., 2011; Escribano-Diaz et al., 2013; Zimmermann et al., 2013); therefore, inhibiting their recruitment to DNA breaks may promote the HR pathway. In line with this hypothesis, depletion of RNF169 reduces HR repair, while over-expression of RNF169 causes increased HR efficiency (Poulsen et al., 2012). A shift toward HR-mediated repair may be favorable since it is less error-prone than the NHEJ pathway.

## DNA DAMAGE-INDUCED SUMOYLATION OF 53BP1 AND BRCA1 BY PIAS1 AND PIAS4

Small ubiquitin-like modifiers and the SUMO conjugation machinery, the E1 SAE1 (SUMO Activating Enzyme E1) and the E2 Ubc9 localize to breaks following damage (Galanty et al., 2009; Morris et al., 2009). Recruitment of SUMO-1 and SUMO-2/3 to DNA breaks is dependent on the SUMO E3 ligases PIAS1 and PIAS4. PIAS1 is specifically required for SUMO-2/3 recruitment while PIAS4, and another SUMO E3 ligase, CBX4, promote recruitment of SUMO-1 and SUMO-2/3 (Galanty et al., 2009; Ismail et al., 2012). Depletion of either PIAS1 or PIAS4 impairs recruitment of BRCA1 (breast cancer 1) and RPA (replication protein A), while depletion of PIAS4 impairs recruitment of 53BP1 (Morris et al., 2009; Galanty et al., 2012). PIAS4 is required for sumoylation of 53BP1 following damage, though the function of this modification remains to be determined. PIAS1 and PIAS4 each promote sumoylation of BRCA1 at K119, which stimulates its ubiquitin ligase activity (Morris et al., 2009). Regulation of the two DSB repair pathways may be mediated by different isoforms of SUMO; depletion of 53BP1 impairs SUMO-1 but not SUMO-2/3 accumulation, while BRCA1 depletion impairs SUMO-2/3 but not SUMO-1 accumulation (Galanty et al., 2009).

## RNF4: LINKING SUMOYLATION AND UBIQUITINATION IN THE DNA DAMAGE RESPONSE

The sumoylation and ubiquitination pathways are directly linked by the E3 RNF4, a member of the SUMO-targeted ubiquitin ligase (STUbL) family that, through four amino-terminal SIMs, preferentially binds and ubiquitinates poly-sumoylated proteins (Prudden et al., 2007; Perry et al., 2008; Tatham et al., 2008). RNF4 has an established role in the DDR, as RNF4 depletion causes increased IR signaling, and impairs RAP80, BRCA1, and RAD51 recruitment to sites of DNA damage (Guzzo et al., 2012; Luo et al., 2012; Vyas et al., 2012; Yin et al., 2012). RNF4 ubiquitinates several sumoylated proteins in the DDR cascade, including MDC1, BRCA1, and RAP80 (Guzzo et al., 2012; Luo et al., 2012; Vyas et al., 2012), and is required for turnover of MDC1 and RPA (Galanty et al., 2012; Luo et al., 2012). Paired with the E2 UbcH5c, RNF4 can catalyze K11-, K48-, or K63-linked ubiquitin chains (Tatham et al., 2008), and at DNA breaks RNF4 contributes specifically to K48 (Luo et al., 2012) and K63 (Yin et al., 2012) linkages.

**FIGURE 3:**
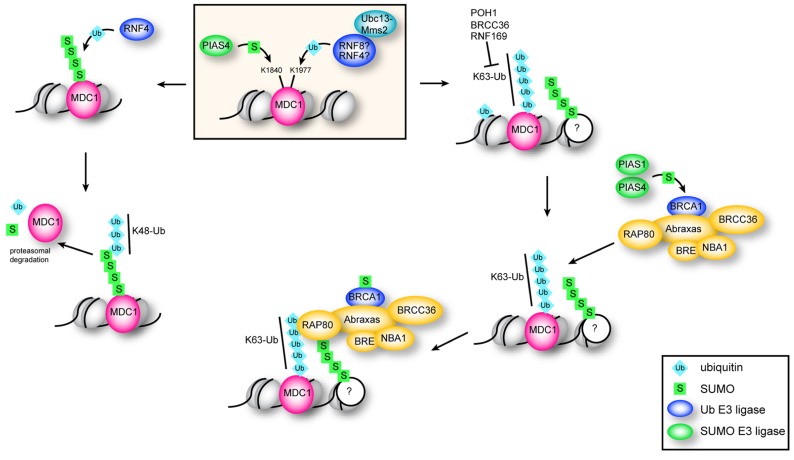
**Function of ubiquitination and sumoylation of MDC1**. Sumoylation of MDC1 at K1840 by PIAS4 recruits the STUbL RNF4. K48 chains catalyzed by RNF4 target sumoylated MDC1 for proteasomal degradation, which is important for efficient HR repair. K63 polyubiquitination of MDC1 at K1977 is dependent on the E2 Ubc13–Mms2 and serves to recruit RAP80. RAP80 accumulates at DSBs through interaction with K63 chains and with sumoylated proteins via its two UIMs and SIM, and is required for efficient recruitment of BRCA1. Sumoylation of BRCA1 by PIAS1 and PIAS4 stimulates its ubiquitin ligase activity.

Although depletion of RNF4 affects both HR and NHEJ, RNF4-mediated ubiquitination of MDC1 specifically impacts the HR pathway of DNA DSB repair by preventing excess accumulation of MDC1 at repair foci (Luo et al., 2012). Following DNA damage, modification of K1840 of MDC1 by SUMO-2/3 recruits RNF4 to sites of DNA breaks (Luo et al., 2012; Yin et al., 2012). MDC1 is then targeted for proteasomal degradation via RNF4-mediated K48-linked ubiquitin chains (Luo et al., 2012; **Figure 3**). Since H2AX/MDC1/53BP1 retention at DNA breaks is antagonistic to HR (Bouwman et al., 2010; Bunting et al., 2010; Helmink et al., 2011), failure to sumoylate MDC1, for example by mutation of K1840, leads to its retention at DNA breaks and inhibition of HR (Luo et al., 2012). MDC1 also mediates recruitment of RAP80 to DNA damage foci. Although the E3 ligase remains unknown, ubiquitination of K1977 within the BRCT domain of MDC1 is required for recruitment of RAP80 to DNA DSBs (Strauss and Goldberg, 2011; Strauss et al., 2011). The K63-specific E2 Ubc13–Mms2 is required for RAP80 recruitment, implying that RAP80 is recruited to breaks through K63-linked ubiquitin chains (Strauss and Goldberg, 2011; Strauss et al., 2011). Specifically, RAP80 is targeted to ubiquitin–SUMO hybrid chains through its SIM and two UIMs, and mutation of either the UIMs or the SIM in RAP80 decreases RAP80 recruitment to repair centers (Guzzo et al., 2012; Hu et al., 2012).

## RNF20–RNF40-MEDIATED H2B UBIQUITINATION: A CRITICAL ROLE IN DNA DSB REPAIR THROUGH CHROMATIN REMODELING

Another important histone modification in DNA repair is the monoubiquitination of histone H2B (ubH2B) on K120, which is an important modification associated with transcriptional elongation in undamaged cells (Xiao et al., 2005). This modification is also required for di-and tri-methylation of K4 and K79 of histone H3 at transcribed chromatin (Kim et al., 2009). At the structural level, ubH2B was shown to interfere with chromatin compaction, leading to an open, more accessible conformation (Fierz et al., 2011). Importantly, the alteration in chromatin structure observed was not simply due to the steric bulk of an added ubiquitin residue. Rather, it was due to intrinsic properties of the modification itself, although the exact residues involved in this chromatin restructuring have yet to be identified. This relaxed conformation may then enhance accessibility of underlying DNA to various transcription factors and their co-regulators.

The E3 ligase responsible for monoubiquitination of H2B is a tight heterodimeric complex of RING-finger proteins RNF20 and RNF40 (Kim et al., 2005; Zhu et al., 2005). Recently, the role of RNF20–RNF40-mediated H2B monoubiquitination in DNA DSB repair has been investigated in several studies (Chernikova et al., 2010; Moyal et al., 2011; Nakamura et al., 2011). These studies demonstrate that monoubiquitination of H2B is required for timely break repair, as abrogation of ubH2B by either RNF20–RNF40 knock-down or over-expression of a non-ubiquitinatable H2B point mutant leads to increased sensitivity to DNA damaging agents with a subsequent reduction in DSB repair efficiency. In addition, when transcription inhibitors are used to reduce the effect of transcription-associated H2B ubiquitination, an elevation of ubH2B can be observed following the induction of DNA DSBs. Notably, a fraction of RNF20–RNF40 was also found to be recruited to DNA DSBs following DNA damage and to interact with ATM and NBS1 (Chernikova et al., 2010; Moyal et al., 2011). Not only does RNF20–RNF40 physically interact with ATM but it also undergoes ATM-mediated phosphorylation, which appears to be required for damage-induced H2B monoubiquitination. However, RNF20 depletion did not affect DNA damage-induced phosphorylation and ubiquitination of H2AX. In fact, several proteins that are recruited via γ-H2AX to break sites such as 53BP1, ATM and MDC1 still formed normal foci in RNF20-depleted cells (Nakamura et al., 2011) suggesting that the RNF20–RNF40 pathway functions independently and/or in parallel to the γ-H2AX-mediated DDR cascade. Collectively these studies demonstrated that ubH2B is not required for the recruitment of damage sensors in early stages of the DDR but is essential for the accumulation of DNA DSB repair proteins involved in both NHEJ (XRCC4 and KU80) and HR (RAD51, RPA, and BRCA1) at DSBs. In addition, both NHEJ and HR repair pathways display retarded repair kinetics when H2B monoubiquitination is abrogated.

Due to the requirement of H2B monoubiquitination for H3K4 and H3K79 methylation during transcription, Moyal et al. (2011) and Nakamura et al. (2011) examined whether ubH2B-dependent methylation at these sites also occur in response to DSBs. While Moyal et al. (2011) did not observe significant differences in methylation, Nakamura et al. (2011) demonstrated that depletion of RNF20 significantly reduces H3K4 and H3K79 methylation following DSB induction. In addition, they noted that SNF2h (sucrose non-fermenting 2 homolog), a subunit of the ATP-dependent chromatin-remodeling complex ISWI that is recruited to sites of transcription through an interaction with methylated H3K4, is recruited to DSBs in an RNF20–RNF40-dependent manner. SNF2h depletion leads to reduced DSB repair through the HR pathway suggesting that chromatin-remodeling mediated by SNF2h influenced repair efficiency. To further support this notion, Nakamura et al. (2011) demonstrated that treatment with several agents that induced chromatin relaxation counteracts RNF20 defects in DNA DSB repair. These experiments suggest that monoubiquitination of H2B by RNF20–RN40 facilitates chromatin decondensation, possibly through SNF2h-mediated chromatin remodeling, so that repair proteins can access the underlying DNA. Since H3K79 methylation by DOT1L and binding of RAD9 via its Tudor domain is also required for efficient single-stranded DNA generation and HR (Lazzaro et al., 2008), changes in H3K79 methylation rather than chromatin-remodeling *per se* may be responsible for the observed defects in DNA DSB repair associated with depletion of RNF20.

## CHROMATIN REMODELING-ASSISTED UBIQUITINATION IN THE DSB RESPONSE

As has been discussed above, ubiquitination can lead to chromatin structural rearrangements in response to DSBs. However, there is evidence that ubiquitin-independent chromatin-remodeling can also facilitate ubiquitination at DSBs, termed chromatin remodeling-assisted ubiquitination. For example, one study recently demonstrated a role for RNF8 in DNA repair that does not depend on its catalytic activity (Luijsterburg et al., 2012). RNF8 was found to recruit the ATPase CHD4 of the nucleosome-remodeling and deacetylase (NuRD) chromatin-remodeling complex to DNA repair foci, rendering DNA more amenable to ubiquitination (Denslow and Wade, 2007; Luijsterburg et al., 2012). Lack of CHD4 activity led to decreased ubiquitination at DSBs and consequently, defective BRCA1 recruitment (Luijsterburg et al., 2012). The authors demonstrate that CHD4 is required for efficient ubiquitination of chromatin, as RNF8 is only briefly associated with chromatin (Mailand et al., 2007) and artificially prolonging RNF8 retention at chromatin bypassed the need for CHD4. The authors propose that RNF8-mediated CHD4 recruitment, and subsequent chromatin decondensation could create a more amenable local chromatin environment for ubiquitination by promoting RNF168 and BRCA1 assembly.

Another study describes a role for the p400 ATPase (a component of the mammalian NuA4 complex) in regulating nucleosome stability and RNF8-mediated chromatin ubiquitination in DNA DSB repair (Xu et al., 2010). DNA damage destabilizes nucleosomes within chromatin regions surrounding DNA DSBs in an active process requiring the ATPase activity of p400, in addition to the histone acetylation activity of the acetyltransferase Tip60. p400 was found to be recruited to DNA DSBs through interaction with MDC1, which was independent of ATM phosphorylation. Interestingly, suppression of RNF8 did not affect the p400-mediated decrease in nucleosome stability at DNA DSBs, indicating that RNF8 ubiquitination does not contribute to p400 chromatin-remodeling activity. However, RNF8-dependent ubiquitination and the subsequent recruitment of BRCA1 and 53BP1 at DNA DSBs required nucleosome destabilization by p400. The authors propose a model whereby DSB induction leads to the generation of γ-H2AX and subsequently the recruitment of MDC1. Components of the NuA4 complex, importantly p400 and Tip60 are recruited to breaks through MDC1, and the ATPase activity of p400 in conjunction with Tip60 histone acetylation then disrupts local chromatin structure leading to a more open, relaxed conformation. This open conformation exposes RNF8 ubiquitination targets as well as histone methylation sites such as H4K20me2, facilitating recruitment of PIAS1/PIAS4, BRCA1, and 53BP1 to DNA DSBs.

Here we have described two different instances of chromatin remodeling-assisted ubiquitination involving RNF8: one involving CHD4 of the NuRD complex, and the other the ATPase p400 of NuA4. It is clear that multiple chromatin-remodeling events take place in response to DNA DSBs. Whether they all function simultaneously or are evoked in response to different stimuli to mediate alternative repair pathways (NHEJ or HR for instance) remains to be determined. Deciphering the exact mechanism involved in DNA DSB-induced chromatin restructuring represents a challenge for future studies.

## SUMOYLATION OF THE KRAB DOMAIN-ASSOCIATED PROTEIN 1 AND THE REPAIR OF DNA BREAKS WITHIN HETEROCHROMATIN

Post-translational modification of many transcription factors or cofactors by sumoylation is generally associated with transcriptional repression (Verger et al., 2003). SUMO modification provides binding sites for diverse chromatin-remodeling enzymes and chromatin-associated proteins such as histone deacetylase 2 (HDAC2), histone demethylase LSD1, heterochromatin protein 1 (HP1), and the NuRD complex that subsequently mediate chromatin compaction and gene silencing (Ouyang and Gill, 2009).

Sumoylation of the transcriptional co-repressor KAP1 (KRAB domain-associated protein 1) is involved in the maintenance of heterochromatin structure. KAP1 is an SUMO E3 ligase, which undergoes auto-sumoylation (Ivanov et al., 2007) and directly interacts with the NuRD complex (Schultz et al., 2001), promoting ATP-dependent chromatin compaction in heterochromatin. NuRD is a multi-subunit complex that couples ATPase chromatin-remodeling activities (through Mi-2 proteins CHD3 and CHD4) with histone deacetylation (through HDAC1/HDAC2 subunits; Goodarzi et al., 2011). The interaction between KAP1 and the NuRD complex is mediated by the CHD3 component, which contains a SIM at its carboxy-terminus. Due to its role in chromatin compaction, KAP1 poses a substantial barrier to DNA DSB repair in heterochromatin. In order for effective repair to occur within heterochromatin, dynamic alterations to chromatin structure are required. Phosphorylation of KAP1 (pKAP1) on S824 by ATM has been shown to be essential for DSB repair in heterochromatic regions (Goodarzi et al., 2008), and to enhance cellular survival following IR (Ziv et al., 2006; Noon et al., 2010).

A recent study put forth a mechanism of pKAP1-mediated chromatin relaxation and heterochromatic DSB repair (Goodarzi et al., 2011). Following IR, ATM induces pKAP1, resulting in dispersion of CHD3 from DNA DSBs, and also triggering a relaxation of chromatin structure. Importantly, CHD3 depletion alleviated repair defects caused by inhibition of ATM or the expression of a non-phosphorylatable S824A KAP1 mutant. CHD3 activity is targeted to KAP1 through interactions between its SIM domain and sumoylated KAP1, and consequently ablation of this interaction by expression of KAP1 with mutated SUMO conjugation sites bypasses the role of pKAP1 in repair. Collectively this data suggests that CHD3 activity associated with sumoylated KAP1 is inhibitory to DSB repair; however, this effect can be alleviated by ATM-mediated pKAP1.

Two possible scenarios can be envisaged for how CHD3 mediates chromatin structural changes in KAP1-dependent heterochromatin following DNA damage. First, CHD3 activity could affect sumoylated KAP1 levels; however, levels of KAP1-SUMO are not altered upon DSB induction. Alternatively, DSB-induced pKAP1 might directly interfere with the interaction between CHD3 and KAP1-SUMO. Consistent with this theory, reduced amounts of CHD3 were observed to interact with phosphomimetic KAP1 following IR. Goodarzi et al. (2011) postulate that DNA damage-induced pKAP1 increases negative charge at the carboxy-terminal region of KAP1, effectively interfering with interactions between SUMO conjugated to KAP1 and the SIM domain of CHD3. This would result in the release of CHD3 from KAP1-enriched heterochromatin to relax chromatin structure and facilitate DSB repair.

The regulated dephosphorylation of KAP1 may also play a parallel or additive role in regulating heterochromatin organization during the DDR (Li et al., 2007, 2010). For example, dephosphorylation of pKAP1 at Ser824 by protein phosphatase 1 (PP1) was shown to regulate sumoylation of KAP1 (Li et al., 2010). Importantly, two PP1 isoforms (PP1α and PP1β) were found to differentially interact with KAP1 (PP1α under unstressed conditions and PP1β under genotoxic stress) and to dephosphorylate KAP1 at Ser824. PP1α was found to regulate basal KAP1 dephosphorylation while PP1β played a role in dephosphorylation of KAP1 Ser824 following modification by ATM kinase in response to DNA DSBs. It was postulated that PP1α, which is constitutively associated with KAP1, may serve to set a threshold for the degree of ATM pKAP1 required to overcome S824 dephosphorylation and consequently sumoylation of KAP1 during the DDR. In this model, after DNA repair is complete, PP1α in conjunction with PP1β would then serve to restore KAP1 sumoylation levels, and hence its role in transcriptional repression and the maintenance of heterochromatin structure.

## CONCLUSION AND FUTURE DIRECTIONS

The study of the regulation of the cellular response to DNA damage is a rapidly advancing field. Findings from the last few years have underscored a role for ubiquitin and SUMO in the DDR signaling cascade. While many substrates of sumoylation and ubiquitination have been identified, for many of these target proteins the modification sites have yet to be determined. The next stage in our understanding of the DDR will require identification of individual modification sites in these proteins in order to assign specific functions to each sumoylation and ubiquitination event. Abolishing sumoylation of a single protein in a DDR pathway may not, however, always yield appreciable phenotypes. For example, one recent study demonstrated that in yeast, simultaneous mutation of the sumoylation sites in multiple repair proteins was required to significantly affect the repair of DNA DSBs by the HR pathway (Psakhye and Jentsch, 2012). This study hints to the potential for a high degree of redundancy in the signaling pathways employing UBLs for the regulation of the DDR, with the caveat that the universality of these results cannot be determined until similar studies are completed in other organisms. In addition, other UBLs, such as NEDD8 and ISG15, have also been implicated in the DDR (Desai et al., 2008; Jeon et al., 2012; Blank et al., 2013), which implies a similarly complex networks of E3 ligases and substrates for these UBLs may also exist as a means of controlling the DDR. Therefore, future studies should be directed to investigating the potential role of these other UBL proteins in the DDR, which will further add to our understanding of regulatory post-translational modification networks in the cellular response to DNA DSBs.

There are several gaps in our current understanding of ubiquitin signaling at DNA breaks. For example, the mechanism through which monoubiquitination of H2AX by RNF2 leads to recruitment of ATM has not been elucidated. As highlighted by Mattiroli et al. (2012), the dependence of RNF168 recruitment on the catalytic activity of RNF8 has not yet been explained; therefore, further studies should pursue identification of additional RNF8 substrates. While we now have an understanding of how ubiquitin regulates ejection of JMJD2A and L3MBTL1 from the chromatin docking sites for 53BP1, the extent of overlap of these two pathways is not clear. As well, the predicted UBDs and newly identified SIMs in several members of the BRCA1-A complex (Abraxas, BRCC36, BRE; Guzzo et al., 2012) will need to be assessed for their potential contribution to DNA repair.

In addition, there is mounting evidence that the two major sites of SUMO-1 and SUMO-2/3 accumulation in the cell, the nuclear lamina and promyelocytic leukemia nuclear bodies (PML NBs), may play diverse roles in the DDR. Both of these compartments are enriched in SUMO E3 ligases and STUbLs, including RanBP1 and Slx5–Slx8 (the yeast RNF4 homolog) at the nuclear lamina and PIAS1, PIAS4, and RNF4 in PML NBs (Pichler et al., 2002; Nagai et al., 2011). In particular, it should be noted that PML is sumoylated, contains a SIM, and was one of the first identified substrates of RNF4, which regulates PML degradation in response to arsenic treatment (Dellaire and Bazett-Jones, 2004; Bernardi and Pandolfi, 2007; Lallemand-Breitenbach et al., 2008; Tatham et al., 2008). PML NB number is also regulated by DNA damage through ATM and KAP1 (Dellaire et al., 2006; Kepkay et al., 2011), and these bodies are associated with a host of DNA repair factors and cell cycle checkpoint proteins that shuttle to and from this subnuclear domain in response to DNA DSBs; these include BLM, WRN, NBS1, MRE11, TopBP1, CHK2, and p53, several of which are targets of sumoylation themselves (Dellaire and Bazett-Jones, 2004). Finally, both of these compartments are also associated with “late” DNA repair foci that may indicate unrepaired or difficult to repair DNA DSBs in mammalian cells (Dellaire et al., 2009). In yeast, unrepaired breaks are recruited to the nuclear lamina where they are sequestered as a possible means of inhibiting inappropriate HR (Oza et al., 2009; Lisby et al., 2010) whereas the juxtaposition of DNA breaks at PML NBs in mammalian cells may enhance HR, as depletion of PML impairs the HR pathway of DNA repair (Yeung et al., 2012). Given the multi-faceted association of these compartments with both DNA repair processes and the sumoylation machinery, future studies should look beyond DNA repair foci to consider the role of PML NBs and the nuclear lamina in coordinating the trafficking, post-translational modification and degradation of proteins in the DDR that are subjected to modification by UBLs.

## Conflict of Interest Statement

The authors declare that the research was conducted in the absence of any commercial or financial relationships that could be construed as a potential conflict of interest.
